# An online assessment of pricing for ready‐to‐drink alcohol products: Implications for risk and policy solutions

**DOI:** 10.1111/acer.15491

**Published:** 2024-11-17

**Authors:** Matthew E. Rossheim, Kayla K. Tillett, Viktor Vasilev, Cassidy R. LoParco, Theresa Agwuncha, Vishaldeep K. Sekhon, Edna P. Mendoza, Olivia Townsend, Maria T. Julian, Ryan D. Treffers, Melvin D. Livingston, Michael B. Siegel, David H. Jernigan

**Affiliations:** ^1^ College of Public Health University of North Texas Health Science Center Fort Worth Texas USA; ^2^ Texas College of Osteopathic Medicine University of North Texas Health Science Center Fort Worth Texas USA; ^3^ Milken Institute School of Public Health George Washington University Washington District of Columbia USA; ^4^ Department of Medicine Johns Hopkins University School of Medicine Baltimore Maryland USA; ^5^ Lewis & Clark College Portland Oregon USA; ^6^ CounterTools Chapel Hill North Carolina USA; ^7^ Pacific Institute for Research and Evaluation (PIRE) Santa Cruz California USA; ^8^ Rollins School of Public Health Emory University Atlanta Georgia USA; ^9^ Tufts University School of Medicine Boston Massachusetts USA; ^10^ School of Public Health Boston University Boston Massachusetts USA

**Keywords:** alcohol, marketing, policy, retail surveillance

## Abstract

**Background:**

Alcohol pricing policies can reduce population‐level alcohol consumption. To inform these policies, it is essential to understand the price per standard alcoholic drink of the least expensive brands. This study focused on prices of ready‐to‐drink products because of their accessibility, popularity among young people, and market expansion in recent years.

**Methods:**

In 2023, we systematically identified 39 retail stores selling alcohol online in Fort Worth, Texas. For each product, we recorded information regarding brand name, alcohol‐by‐volume (abv), liquid volume, and price (*n* = 10,818). Ready‐to‐drink products encompassed beer, malt liquor, cider, premixed cocktails, and flavored alcoholic beverages (FAB) including hard beverages (seltzer, soda, tea, lemonade), excluding wine and distilled spirits. We limited analyses to brands sold by at least three stores and deduplicated products within stores. Our analytic sample size was 3924.

**Results:**

The least expensive brands included the following: Four Loko, MXD Drinks Co., Steel Reserve (High Gravity Lager and Alloy Series), Hurricane High Gravity, Natural Ice, Natty Daddy, Clubtails, Sauza Agave Cocktails, Truly Extra, and Icehouse. The average abv among all products was 5.9%. Among the 20 least expensive brands, the average abv was 9.0%, and 70% were available in single‐serve containers.

**Conclusions:**

The least expensive brands of ready‐to‐drink alcohol products were often high abv, single‐serve containers of FAB, malt liquor, or beer. Retail price assessments can strengthen the case for policy solutions, such as targeted taxes and re‐classification of products, to reduce the risks posed by low‐priced alcohol. The current study identifies some brands these retail assessments should include.

## Graphical abstract

This study systematically analyzed the prices of ready‐to‐drink alcohol products sold online in Fort Worth, Texas. It identified the least expensive brands, which were often high alcohol content, single‐serve containers of flavored alcoholic beverages, malt liquor, or beer. The findings highlight the need for policy interventions, such as taxes or alcohol content restrictions, to mitigate public health risks. Local retail price assessments can further support these efforts, and this study identifies key brands that should be prioritized in such assessments.

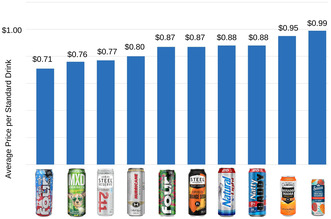

## INTRODUCTION

In the United States (U.S.), more than 140,000 people die each year from excessive alcohol use (Esser et al., 2024; Pilar et al., 2020). Alcohol pricing policies are some of the most effective population‐level strategies for reducing alcohol‐related morbidity and mortality (Wagenaar et al., 2010; World Health Organization, 2017). Strong epidemiological evidence shows that higher alcohol prices are associated with significantly less alcohol consumption as well as related violence, crime, sexually transmitted infections, and deaths (Wagenaar et al., 2009, 2010). Notably, young people and those who drink heavily are especially responsive to price changes (Elder et al., 2010; Shrestha, 2015).

Various pricing policy options, including excise taxes and minimum unit pricing (MUP) laws, are available at the federal, state, and (unless preempted by state laws) local level. The impact of alcohol pricing laws largely depends on the current prices of alcohol products, particularly the least expensive products of each type. Thus, retail price assessments are crucial for understanding the potential impact that various pricing laws may have on alcohol prices and, therefore, consumption.

In conducting these retail price assessments, it is essential to account for the significant variability in alcohol content across different products. Thus, the concept of a “standard alcoholic drink” (i.e., 0.6 ounces of pure alcohol) emerges as a key factor in accurately comparing the cost of various alcohol products. Regardless, identifying the least expensive alcohol products available in physical settings can be challenging due to the wide variety of brands, packaging sizes, liquid volume, and alcohol‐by‐volume (abv) percentages, all of which influence the price per standard alcoholic drink. This complexity is especially evident for ready‐to‐drink alcohol products, which have large variability in their number of standard alcoholic drinks and labeling that does not clearly specify this information (Rossheim, Yurasek, et al., 2019). Therefore, to effectively conduct price assessments in retail settings, it is crucial to determine which brands offer the lowest cost per standard alcoholic drink. Addressing this need, the current study utilizes online data to systematically identify the least expensive ready‐to‐drink alcohol brands.

The most recent U.S. study to systematically examine the prices of inexpensive alcohol brands was conducted in 2011 (DiLoreto et al., 2012). There have since been several major changes to the U.S. alcohol market. From January 2011 to January 2023, there has been 36% inflation, such that $1 in 2011 has the same purchasing power as $1.36 in 2023 (U.S. Bureau of Labor Statistics, 2023). Thus, we would expect the prices of all alcohol products to have risen during this time. However, at least three other major changes may have influenced alcohol prices since 2011: (a) the expansion of the ready‐to‐drink alcohol category including hard seltzers, sodas, coffee, kombucha, lemonade, tea, and canned cocktails, (b) the increased total alcohol content of ready‐to‐drink products (including Four Loko, Smirnoff Ice Smash, and Mike's Harder Lemonade), and (c) the evolving online alcohol market and rapid expansion of online retail commerce, likely due in part to the COVID‐19 pandemic, during which U.S. drinking rates also increased (Grossman et al., 2020; Plata et al., 2022).

The current study used methods validated in previous research to conduct an online retail price assessment of ready‐to‐drink alcohol products (Colbert et al., 2020; DiLoreto et al., 2012; Williams & Schmidt, 2014). We focused on ready‐to‐drink products for four reasons: (a) to test the feasibility of these methods in the current online environment, (b) because only an off‐premise beer license is needed to sell these products, so they are sold at any off‐premise retailer (not just liquor stores), (c) because products in this category tend to be popular among underage drinkers (Fortunato et al., 2014; Rossheim, Greene, et al., 2019), and (d) because the ready‐to‐drink market has had some of the most notable changes regarding product categories and alcohol content since 2011. Moreover, online U.S. retail is especially important to study because the lack of effective age verification practices, making alcohol highly accessible to minors (Williams & Ribisl, 2012; Williams & Schmidt, 2014).

Thus, in the current study, we examined the prices of ready‐to‐drink products listed online by retail stores located in Fort Worth, Texas. We aimed to identify and describe the least expensive ready‐to‐drink alcohol brands, including their alcohol content and price per standard drink. For this study, the term “brand” refers to the name on the product's label that identifies the specific type of alcohol being marketed and sold.

## MATERIALS AND METHODS

### Study overview

The current study is a cross‐sectional assessment of online alcohol retail products and prices available for pickup and delivery in Fort Worth, Texas. Data collection was focused on a single city to ensure consistent excise taxes. Fort Worth was an ideal focal point due to its large and diverse population, blend of urban and suburban settings, and wide range of consumer preferences, fostering a variety of retail environments and competition (CBRE, 2023).

### Store sample

The study's methods, including search terms, were informed by previous research (Colbert et al., 2020; DiLoreto et al., 2012; Williams & Schmidt, 2014). In February 2023, we conducted a Google search for “alcohol fort worth tx.” This search phrase was adapted from previous research (Colbert et al., 2020) with the goal of locating stores in Fort Worth, Texas, as well as assessing what alcohol is generally available, rather than searching for specific types. To reduce bias based on search history, we used Incognito mode, which allows for Internet searches without a record of search history, cookies, or other temporary data. To provide a reasonable depth for our search, we examined the first 100 results and included all websites selling alcohol online to the public. When a street address or ZIP code was required as a shipping address to access the site, we entered information for the lead investigator's university address: 3500 Camp Bowie Blvd, Fort Worth, TX, 76107.

Five stores were excluded from the analysis, because four did not sell alcohol online (nor list prices) and one did not sell ready‐to‐drink products. The remaining links were excluded because they were duplicates or irrelevant to our study as they led to alcohol treatment information, news articles, events, restaurants, job listings, stores listed for sale, legal services, drug testing services, beverage copacking services, or government resources related to alcohol laws or licensing.

Our analytic sample included 39 stores: seven convenience stores (18%), 25 liquor stores (64%), and seven supermarket/grocery stores (18%). After deduplicating websites, there were 13 websites selling alcohol online from 38 different stores in Fort Worth, TX. We also recorded data from the Walmart website for the nearest location because Walmart is a major player in the U.S. retail industry (Fishman, 2006). Walmart is one of the most widespread brick‐and‐mortar retailers across the United States and is one of the world's largest companies in terms of revenue (Fishman, 2006). Moreover, through negotiations, they have some authority in determining the retail price of products; their efforts to maintain “everyday low prices” may drive down alcohol prices (Fishman, 2006).

### Data collection

Data were collected from February to April 2023. Information was recorded for every ready‐to‐drink alcohol product, which encompassed beer, malt liquor, cider, premixed cocktails, and flavored alcoholic beverages (FAB) including hard beverages (e.g., seltzers, sodas, tea, lemonade, kombucha), excluding wine and distilled spirits. We recorded brand names and information used to calculate the price per standard alcoholic drink: liquid volume, abv, and price (including promotions and excluding sales tax and shipping). Brands were categorized based on the name displayed on the packaging. In cases where a single brand offered products with varying abv percentages, such as Four Loko with both 12% and 14% abv options, each variation was treated as a distinct category. Missing abv information was logically imputed based on the same product's abv at other stores in the sample and verified using the manufacturer's website. After training the researchers in data collection, the lead investigator (MER) reviewed a random subset of each researcher's data, confirming very high accuracy across all collected data. There was only one error in data entry from the 200 cases reviewed: a product sold for $1.99 which was mistakenly entered as a 6‐pack rather than as a single unit. This error, and similar errors in data entry, would have also been detected during data cleaning.

### Analytic sample and variables

Data were recorded for 11,196 cases pertaining to ready‐to‐drink alcohol products. We did not record information for “nonalcoholic” drinks, defined in our study as an abv ≤0.5%. Overall, 380 cases (3.4%) were removed from analyses due to missing data on abv (*n* = 296), volume (*n* = 70), and/or price (*n* = 19): 10,816 cases remained.

The number of standard alcoholic drinks in each product was computed by multiplying the percent abv and total volume (in ounces) and dividing by 0.6 ounces (i.e., one standard alcoholic drink). For example, a 12 oz. beer with 5% abv, a 5 oz. glass of wine with 12% abv, and a 1.5 oz. shot with 40% abv each contain one standard alcoholic drink (12*0.05/0.6 = 5*0.12/0.6 = 1.5*0.40/0.6 = 1). Price per standard alcoholic drink was calculated by dividing price by number of standard alcoholic drinks (O'Mara et al., 2009). The current study focused on identifying brands that afforded consumers the most alcohol for the least amount of money. Thus, we systematically deduplicated products, selecting the least expensive option available in each store for each brand. This was necessary because stores often sell different pack sizes (e.g., 1, 4, 6, 8, 12, 15, 18, 24, and 30 packs), container sizes (e.g., 12, 16, and 24 oz), and container types (bottles or cans) for the same brand, each with a different price per standard drink. We deduplicated products of the same brand in the same store in two ways, to create two different analytic samples with equivalent sample sizes: (a) by keeping only the least expensive version per standard alcoholic drink and (b) by keeping only the version that provided the most standard alcoholic drinks for $20 or less. This latter metric was particularly relevant for understanding the brands that can provide the most alcohol to those with limited disposable income, including young people. After deduplicating products, the sample size was 6067.

To focus on products that were widely distributed, we limited our analyses to brands that were sold in at least three stores. The resulting analytic datasets included 3924 cases across 539 brands of ready‐to‐drink alcohol products (Tables 1 and 2). We then aggregated data within each of the 539 brands by calculating the average price per standard drink and the average number of drinks available for $20 for each brand. This allowed us to examine the average abv between brands, grouped by their price levels, as shown in Table 3.

**TABLE 1:** Prices of the least expensive ready‐to‐drink alcohol brands (*n* = 3924).

Rank	Brand type	Alcohol‐by‐volume (%)	Single serve container sizes (oz)	Multicontainer package sizes (containers × oz)	Average price per standard drink (range)
1	Four Loko *Flavored Alcoholic Beverage*	14	23.5	N/A	$0.71 ($0.60–$0.78)
2	MXD Drinks Co *Flavored Alcoholic Beverage*	12	16	N/A	$0.76 ($0.62–$0.90)
3	Steel Reserve *Malt Liquor*	8.1	24 42	4 × 16 6 × 16 12 × 12	$0.77 ($0.59–$0.92)
4	Hurricane High Gravity *Malt Liquor*	8	24	N/A	$0.80 ($0.80–$0.80)
5	Four Loko *Flavored Alcoholic Beverage*	12	23.5	N/A	$0.87 ($0.65–$1.06)
6	Steel Reserve: Alloy Series *Flavored Alcoholic Beverage*	8	24	N/A	$0.87 ($0.65–$1.25)
7	Natural Ice *Beer*	5.9	25	4 × 16 15 × 12	$0.88 ($0.67–$1.13)
8	Natty Daddy *Malt Liquor*	8	25	N/A	$0.88 ($0.84–$0.90)
9	Clubtails *Flavored Alcoholic Beverage*	10	16	4 × 6.76	$0.95 ($0.67–$1.66)
10	Sauza Agave Cocktails *Flavored Alcoholic Beverage*	8	N/A	6 × 12 12 × 12	$0.99 ($0.84–$1.14)
11	Truly Extra *Flavored Alcoholic Beverage*	8	16		$1.00 ($0.93–$1.03)
12	Real Ale *Beer*	8.1	19.2	6 × 12 12 × 12	$1.01 ($0.82–$1.34)
13	Milwaukee's Best Light *Beer*	4.8	24 32	6 × 16 24 × 12	$1.01 ($0.78–$1.29)
14	Icehouse *Beer*	5.5	24	6 × 16	$1.02 ($0.68–$1.31)
15	Bishop *Beer*	8.5	N/A	6 × 12	$1.03 ($0.93–$1.15)
16	Keystone Ice *Beer*	5.9	24	6 × 16	$1.03 ($0.91–$1.27)
17	Karbach *Beer*	9.5	N/A	6 × 12	$1.04 ($0.98–$1.09)
18	Squatters *Beer*	9	N/A	6 × 12	$1.05 ($0.95–$1.22)
19	Tupps *Beer*	12.1	N/A	4 × 12	$1.05 ($0.62–$1.29)
20	Stone Brewing *Beer*	9.4	N/A	6 × 12	$1.05 ($0.93–$1.15)

**TABLE 2:** Ready‐to‐drink brands that afforded the most alcohol for $20 (*n* = 3924).

Rank	Brand	Alcohol‐by‐volume (%)	Single serve container sizes (oz)	Multi‐container package sizes (containers × oz)	Average standard drinks for $20
1	Four Loko *Flavored Alcoholic Beverage*	14	23.5	N/A	25.8
2	MXD Drinks Co *Flavored Alcoholic Beverage*	12	16	N/A	25.0
3	Steel Reserve *Malt Liquor*	8.1	24 42	4 × 16 6 × 16 12 × 12	23.3
4	Hurricane High Gravity *Malt Liquor*	8	24	N/A	22.7
5	Steel Reserve: Alloy Series *Flavored Alcoholic Beverage*	8	24	N/A	22.4
6	Clubtails *Flavored Alcoholic Beverage*	10	16	4 × 6.76	21.2
7	Four Loko *Flavored Alcoholic Beverage*	12	23.5	N/A	21.2
8	Natty Daddy *Malt Liquor*	8	25	N/A	20.8
9	Truly Extra *Flavored Alcoholic Beverage*	8	16	N/A	19.9
10	Icehouse *Beer*	5.5	24	6 × 16	19.8
11	Natural Ice *Beer*	5.9	25	4 × 16 15 × 12	19.3
12	Keystone Ice *Beer*	5.9	24	6 × 16	18.9
13	Mickey's *Malt Liquor*	5.6	24 40	6 × 12	17.9
14	Olde English *Malt Liquor*	5.9	24 42	N/A	16.9
15	Real Ale *Beer*	8.1	19.2	6 × 12 12 × 12	16.7
16	Neon Burst *Flavored Alcoholic Beverage*	8.0	25	N/A	16.3
17	Tupps *Beer*	12.1	N/A	4 × 12	16.1
18	Mike's Harder *Flavored Alcoholic Beverage*	8	16 23.5	8 × 16 12 × 11.2	16.0
19	Sauza Agave Cocktails *Flavored Alcoholic Beverage*	8	24	6 × 12 12 × 12	16.0
20	Redd's *Flavored Alcoholic Beverage*	8	24	N/A	16.0

**TABLE 3:** Average ABV of ready‐to‐drink products across average price groups (*n* = 539 brands).

Price percentile range	Grouped by average price per standard alcoholic drink		Grouped by average number of drinks available for $20	
	Average price per standard drink	Average alcohol‐by‐volume	Average number of drinks for $20	Average alcohol‐by‐volume
0–1	$0.71–$0.87	10.8	21.2–25.8	10.0
1–5	$0.87–$1.08	9.4	15.2–21.2	7.8
5–10	$1.08–$1.18	8.2	13.0–15.2	7.6
0–10	$0.71–$1.18	8.9	13.0–25.8	7.9
10–20	$1.18–$1.37	7.6	11.0–12.9	7.6
20–30	$1.38–$1.50	7.6	9.8–10.9	6.9
30–40	$1.50–$1.63	6.2	8.8–9.8	6.1
40–50	$1.63–$1.75	5.8	8.2–8.8	6.0
50–60	$1.75–$1.87	5.8	7.6–8.2	5.9
60–70	$1.87–$2.00	5.7	7.0–7.6	5.7
70–80	$2.00–$2.16	5.1	6.5–7.0	5.5
80–90	$2.16–$2.55	6.4	5.3–6.5	5.7
90–100	$2.56–$12.73	6.2	0–5.3	7.2

## RESULTS

Table 1 displays the 20 least expensive ready‐to‐drink alcohol brands. The average price per standard alcoholic drink in the overall sample was $1.79. Price per standard drink for the least expensive 20 brands ranged from $0.71 to $1.05. The average abv of all products in the analytic sample was 5.9%, whereas the average abv of the 20 least expensive products was 9%, with 80% (16/20) having an abv of 8% or higher. Among the 20 least expensive brands, 70% were available in single‐serve containers. These 20 least expensive brands included seven FABs, three malt liquors, and 10 beers. Brands that afforded notably inexpensive alcohol but were only sold in two stores in the sample included Earthquake (10% abv) and Icehouse Edge (8% abv), costing an average of $0.65 and $0.84 per standard drink, respectively.

Table 2 displays the 20 ready‐to‐drink brands that afforded the most standard alcoholic drinks for $20. Products that afforded the most alcohol for $20 tended to be single‐serve containers (95%) of FAB (50%), malt liquor (25%), or beer (20%). Among these 20 brands, 75% had an abv of 8% or higher.

The top 11 brands were extreme outliers (>3 standard deviations above the mean) in terms of the number of standard drinks they provided for $20. These brands provided 19.3 to 25.8 standard alcoholic drinks for $20. In comparison, the average number of standard alcoholic drinks obtainable for $20 among products in the entire analytic sample was 9.2 (less than half). There was considerable overlap (*n* = 15) between the 20 least expensive brands per standard drink and the 20 brands offering the most standard alcoholic drinks for $20.

To provide further insights regarding whether these patterns persisted across all brands, we conducted additional analyses using aggregate data for each brand (*n* = 539). Table 3 shows the average abv of products that are grouped according to (a) their average price per standard alcoholic drink and (b) the average number of drinks they provide for $20. This table illustrates that the brands with lower price per standard drink and that afford more alcohol for $20 tend to be products with higher abv. For example, the least expensive 10% of brands per standard alcoholic drink ($0.71–$1.18) had an average abv of 8.9% compared to 6.2% for the most expensive 10% of brands ($2.56–$12.73 per standard alcoholic drink). This difference is even more pronounced when looking at a more granular level. For example, the least expensive 1% of brands had an average abv of 10.8%. A similar pattern was observed for average standard drinks available for $20; brands that afforded more alcohol had higher abv.

## DISCUSSION

This study presents current information on the least expensive brands of ready‐to‐drink alcohol products sold online in Fort Worth, Texas. These findings are timely given the recent growth of the FAB category. Notably, brands such as Four Loko, Steel Reserve, and Hurricane remain among the least expensive options since 2011, despite public health appeals (DiLoreto et al., 2012; Rossheim, Thombs, et al., 2019; State Attorneys General, 2015). Meanwhile, several FAB brands introduced post‐2011, including MXD Drinks Co., Truly Extra, Clubtails, Steel Reserve Alloy Series, Sauza Agave Cocktails, and Neon Burst, are now among the least expensive ready‐to‐drink products. The emergence of these new, low‐cost FABs raises significant concerns given their potential to increase consumption and related negative outcomes, especially among young people.

The least expensive products and those that afforded the most alcohol for $20 were predominantly high‐abv FAB or malt liquor and sold in large, single‐serving containers. This is important to note, because state laws often hinder local price intervention through direct taxation (Mosher et al., 2017). However, other local measures, such as regulating abv, liquid volume, types of products, number of containers, or specific brands, may be viable options to indirectly control prices. Evidence from various localities suggests that these types of regulations on malt liquor were associated with modest reductions in crime (Calvert et al., 2020; Jones‐Webb et al., 2021; McKee et al., 2017). Given these findings, communities should consider adopting similar ordinances. The current study underscores the importance of more research on indirect price control strategies and their role in mitigating alcohol‐related problems.

The 14% abv Four Loko, introduced in 2015 (YouTube, 2015), exemplifies the ongoing challenge faced by public health efforts. It is now the least expensive ready‐to‐drink alcohol product, despite an appeal in 2015 by 17 State Attorneys General for the manufacturer to reduce the abv to 8% (State Attorneys General, 2015). Research suggests that capping the alcohol content at 8% abv could mitigate alcohol‐related harms among young people (Rossheim, Greene, et al., 2019). The persistently high alcohol content and affordability of such products, despite notable public health consequences experienced by underage drinkers (Rossheim et al., 2021, 2023; Rossheim, Greene, et al., 2019), underscores the urgent need for enhanced regulations.

### Strengths and limitations

Leveraging data from online retailers enabled the calculation of price per standard alcoholic drink for all major ready‐to‐drink brands, a method impractical in physical retail settings. These insights are vital for community‐led retail price assessments. Our focus on a single city with consistent excise tax ensured that, despite price variations between states, the relative affordability of products remained comparable. Future research should examine the retail price of these brands in other U.S. markets. A UK study corroborates the utility of online data in reflecting in‐store prices (Bhatnagar et al., 2021). However, the potential for additional delivery costs and inability to account for in‐store promotions are study limitations that highlight the value of local data collection. Our comprehensive approach to price assessment offers invaluable data for these initiatives.

## CONCLUSIONS

This research highlights a concerning trend: the persistent low price of certain ready‐to‐drink brands, along with many new, inexpensive FABs. A relatively small number of high‐abv FAB, malt liquor, and beer brands sold in the United States are extreme outliers in terms of their low prices. While state preemption laws may restrict local tax policies, other local regulatory strategies including limiting abv and container sizes have shown promise and merit further exploration (Calvert et al., 2020; Jones‐Webb et al., 2021; McKee et al., 2017). Study findings emphasize the critical need for ongoing research and policy advocacy to confront the issue of inexpensive, high‐abv alcohol products. Such efforts are especially important to protect community health and to prevent the disproportionate impact these products have on young people (Rossheim et al., 2021, 2023; Rossheim, Greene, et al., 2019).

## CONFLICT OF INTEREST STATEMENT

The authors declare no conflicts of interest.

## Data Availability

The data that support the findings of this study are available from the corresponding author upon reasonable request.
